# Clinical Relevance of Different Loads of Perivascular Spaces According to Their Localization in Patients with a Recent Small Subcortical Infarct

**DOI:** 10.3390/jcdd11110345

**Published:** 2024-11-01

**Authors:** Caterina Sozzi, Carla Brenlla, Inés Bartolomé, Andrés Girona, Emma Muñoz-Moreno, Carlos Laredo, Alejandro Rodríguez-Vázquez, Antonio Doncel-Moriano, Salvatore Rudilosso, Ángel Chamorro

**Affiliations:** 1 Neurology Department, University of Milano Bicocca, 20126 Milan, Italy; c.sozzi4@campus.unimib.it; 2 Neurology Department, Hospital Clínic, 08036 Barcelona, Spaingirona@clinic.cat (A.G.); 3 August Pi i Sunyer Biomedical Research Institute (IDIBAPS), 08036 Barcelona, Spain; 4 Comprehensive Stroke Center, Department of Neuroscience, Hospital Clínic, 08036 Barcelona, Spain; alrodriguez@clinic.cat (A.R.-V.);; 5 Faculty of Medicine and Health Sciences, University of Barcelona, 08036 Barcelona, Spain

**Keywords:** cerebral small vessel disease, lacunar stroke, perivascular spaces, sleep

## Abstract

**Background and Purpose**: Perivascular spaces (PVS) are usually enlarged in small vessel disease (SVD). However, the significance of PVS patterns in different locations is uncertain. Hence, we analyzed the distribution of PVS in patients with a recent small subcortical infarct (RSSI) and their correlation with clinical and imaging factors. **Materials and Methods**: In a cohort of 71 patients with an RSSI with complete clinical data, including the Pittsburgh Sleep Quality Index (PSQI), we segmented PVS in white matter (WM-PVS), basal ganglia (BG-PVS), and brainstems (BS-PVS) on 3T-MRI T2-weighted sequences, obtaining fractional volumes (%), and calculated the WM/BG-PVS ratio. We analyzed the Pearson’s correlation coefficients between PVS regional loads. We used normalized PVS measures to assess the associations with clinical and MRI-SVD features (white matter hyperintensities (WMHs), number of lacunes, and microbleeds) in univariable and multivariable linear regressions adjusted for age, sex, and hypertension. **Results**: In our cohort (mean age 70 years; 27% female), the Pearson’s correlation coefficients between WM-PVS/BG-PVS, WM-PVS/BS-PVS, and BG-PVS/BS-PVS were 0.67, 0.61, and 0.59 (all *p* < 0.001). In the adjusted models, BG-PVS were associated with lacunes (*p* = 0.034), WMHs (*p* = 0.006), and microbleeds (*p* = 0.017); WM-PVS with lacunes (*p* = 0.003); while BS-PVS showed no associations. The WM/BG-PVS ratio was associated with lacunes (*p* = 0.018) and the PSQI (*p* = 0.046). **Conclusions**: PVS burdens in different regions are highly correlated in patients with RSSI but with different SVD patterns. Sleep quality impairment might affect waste removal mechanisms differently in the WM and BG regions.

## 1. Introduction

Cerebral small vessel disease (SVD) is an age-related condition highly associated with hypertensive arteriosclerosis that accounts for approximately one-fourth of all acute ischemic strokes, mainly in the form of lacunar infarction [1], and is the most prevalent cause of vascular dementia [2]. Several brain magnetic resonance imaging (MRI) markers have emerged as markers of SVD, such as recent small subcortical infarcts (RSSIs), lacunes, microbleeds, white matter hyperintensities (WMHs), and enlarged perivascular spaces (PVS) [3]. PVS have recently gained interest as markers of SVD, potentially reflecting the early stages of the disease involving endothelial dysfunction [4] and fluid clearance mechanisms (i.e., the glymphatic system) imbalance [5]. PVS on MRIs increase in number with age, vascular risk factors (particularly arterial hypertension), and other features of SVD, indicating that they are clinically relevant [6]. Nevertheless, data in the literature are often heterogeneous, particularly when it comes to identifying patterns of enlarged PVS based on different brain regions and their clinical relevance. In addition, increased visibility of PVS is not specific to sporadic SVD, since they have been related to other neurological diseases such as cerebral amyloid angiopathy and sleep disorders [7,8,9].

This study aims to describe the PVS distribution in patients with an RSSI and analyze their correlations with clinical and imaging features. By achieving this, we aim to provide insights into the clinical relevance of different PVS patterns, including their associations with other SVD markers and sleep quality.

## 2. Materials and Methods

### 2.1. Patients

This is a post hoc analysis including patients from two observational prospective cohorts aimed at studying the relationships between the diffusivity of brain fluids and SVD signatures in patients with an RSSI. One cohort is composed of 50 patients with an RSSI confirmed on MRI. The other one is composed of patients with an RSSI from an ongoing, prospective project. The study protocol was approved by the local Ethics Committee (HCB/2024/0683).

RSSI was defined on MRI according to the Standards for ReportIng Vascular changes on nEuroimaging (STRIVE) criteria [3]. The exclusion criteria were contraindications for MRI, severe dependency at enrollment (modified Rankin Scale > 3), and the presence of old brain lesions that may hamper the interpretation of the results (i.e., brain tumors, prior large vessel strokes or hemorrhages, etc.). The participants underwent an Investigational 3-Tesla MRI within 2 months after the index stroke. The only inclusion criterion for this substudy implied evaluable MRI sequences for PVS assessment (T2-weighted sequences). The collected variables included age, sex, body mass index, smoking and drinking habits, history of arterial hypertension, diabetes, hyperlipidemia, atrial fibrillation, National Institute of Health Stroke Scale (NIHSS) at admission, and sleep quality questionnaires (Epworth Sleepiness Scale (ESS) and Pittsburgh Sleep Quality Index (PSQI)).

### 2.2. Investigational MRI Acquisition

All MRI studies were acquired at the IDIBAPS MRI Core Facility with a 3T MRI scanner (MAGNETOM Prisma, Siemens Healthineers, Erlangen, Germany) with a 32-channel phased-array coil. We established an image acquisition protocol according to the HARNESS recommendations for the clinical research on SVD [10], including the following sequences: 3D T1-weighted (1.0 × 1.0 × 1.0 mm^3^, matrix 256 × 256, FOV 256 × 256 mm^2^, number of slices 192, TR/TE/TI 2500/4.37/1100 ms); 3D T2-weighted (0.9 × 0.9 × 0.9 mm^3^, matrix 256 × 256, FOV 240 × 240 mm^2^, number of slices 176, TR/TE 3200/406 ms); 3D Fluid-Attenuated Inversion Recovery (FLAIR, 0.5 × 0.5 × 1.0 mm^3^, matrix 512 × 512, FOV 256 × 256 mm^2^, number of slices 192, TR/TE/TI 5000/388/1800 ms); Susceptibility-Weighted Imaging (SWI, 0.6 × 0.6 × 3 mm^3^, matrix 312 × 384, FOV 195 × 240, number of slices 52, TR/TE 28/20 ms); multi-shell diffusion-weighted imaging (DWI, 2.0 × 2.0 × 2.0 mm^3^, matrix 112 × 112, FOV 224 × 224 mm^2^, number of slices 76, TR/TE 3000/113 ms, 14 baseline images (b-value = 0) and three shells with b-values 500 s/mm^2^, 1000 s/mm^2^, 2000 s/mm^2^ and 6, 64, and 64 gradient directions, respectively). A technician checked the quality of each sequence during acquisition and repeated the specific sequence acquisition when it was not valuable for movement artifacts or other reasons.

### 2.3. SVD Assessment on MRI

**Figure 1:**
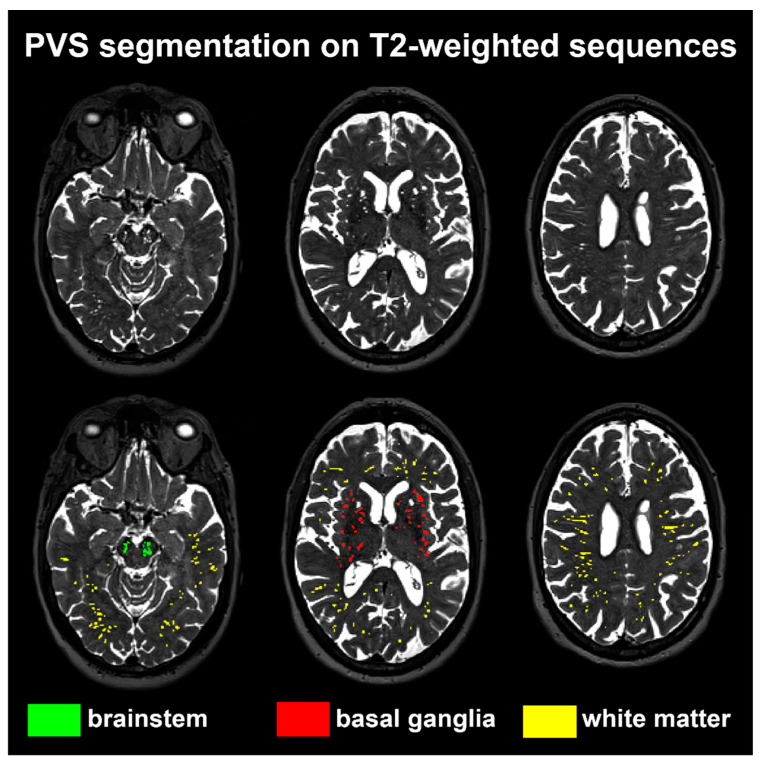
PVS regional segmentation. This image shows the results of the PVS segmentation method from a case. The top row displays the raw T2-weighted sequence images at the midbrain, basal ganglia, and centrum semiovale levels. The PVS in the brainstem are labeled in green, those in the basal ganglia in red, and those in the white matter in yellow. It is noteworthy that the PVS segmentations do not precisely correspond to the region of interest of the visual score. For instance, PVS in the white matter were also segmented in the temporal lobes, as illustrated in the figure.

The radiological variables were already available from the main study datasets. The SVD markers were defined according to the STRIVE criteria [3]. Lacunes were identified and counted on T1/T2 sequences; WMHs were evaluated in periventricular and deep white matter areas on FLAIR sequences with a rating ranging from 0 to 3 points according to the Fazekas score [11]; cerebral microbleeds were identified on SWI sequences. The load of visible PVS on T2-weighted sequences was scored according to a 5-point scale in the basal ganglia and centrum semiovale [12]. The computational segmentation of PVS was performed using a published, validated technique based on the 3D Frangi filter to enhance vessel-like structures [13,14]. In normal-appearing white matter (NAWM), the Frangi filter was applied to the T2-weighted image, while in the areas of WMHs, both T2-weighted and FLAIR images were used to improve the detection of PVS in WMHs and avoid false positive segmentations. The resulting imaging was then thresholded to identify the PVS. The threshold was chosen using the visual rating as a reference and applied to the whole cohort. To improve the segmentation accuracy, we applied specific filter parameters and thresholds for NAWM and WMHs [15] and applied a filter removing small clusters of 5 or fewer voxels (3.65 mm^3^), mostly due to background noise. We also removed the areas including lacunes and the subacute stroke. We selected three regions for PVS quantification, defined as white matter (WM-PVS), basal ganglia (BG-PVS), and brainstems (BS-PVS). The regions of interest segmentation was performed with the Freesurfer image analysis suite (http://surfer.nmr.mgh.harvard.edu/). The WM region included frontal, parietal, temporal, and occipital lobes, excluding fiber tracts between the basal ganglia. BG region refers to deep gray structures, including the lenticular nucleus, caudate, thalamus, and internal and external capsule. The BS region refers to the medulla, pons, and midbrain. We obtained computational measures of the fractional PVS volume standardized for the volumes (%) of the basal BG, WM, and BS, respectively [14,16]. We also obtained the ratio of fractional PVS volume between the WM and BG. An example of PVS segmentation is shown in Figure 1. We segmented the WMHs using an automated pipeline [14]. The subacute stroke lesion volume was removed after manual segmentation using ITK-SNAP v. 3.8.0 software to avoid erroneous identification as WMHs. WMH volume was normalized by intracranial volume (WMH/ICV, %). All PVS and WMH segmentations were checked by a neurologist (SR) and a neurology resident (CS), who edited them manually if required. The total brain volume (gray and white matter excluding ventricles) was obtained by FreeSurfer quantification.

### 2.4. Statistical Analysis

Means or medians were used as central tendency and standard deviation measures, and interquartile ranges as dispersion measures according to each variable’s parametric or non-parametric features. Using ANOVA and the Kruskal–Wallis test, respectively, we analyzed the variance between the means and medians of the clinical, demographic, and radiological variables in the different quartiles of BG-PVS, WM-PVS, and BS-PVS.

We used Spearman’s correlation coefficient for the analysis of non-parametric variables—that is, the correlations between PVS visual scores and fractional volumes according to each region (BG, WM, and BS). With Pearson’s coefficient, applied to parametric variables, we analyzed the correlation between fractional volumes of WM-PVS, BG-PVS, and BS-PVS. To study the association between the burden of PVS in each region and clinical, demographic, and radiological variables, we performed linear regression analyses. For this purpose, we tested 9 different variable transformations (cubic, square, identity, square root, logarithmic, 1/square root, inverse, 1/square, and 1/cubic) of the dependent variable (PVS fractional volume) to achieve a normal distribution and chose the one that fitted better for the 3 regions. We used linear unadjusted regression analysis and then a model adjusted by age and history of arterial hypertension as pre-established relevant confounders. To assess the impact of the PVS differential load between the WM and BG regions, we finally performed the same univariate and multivariate analyses by using the WM/BG-PVS ratio as the dependent variable.

We considered statistically significant values of *p* < 0.05 for all analyses, and all hypotheses were 2-sided. Analyses were performed in Stata v18.0 for Windows 11 (College Station, TX, USA: StataCorp LLC).

## 3. Results

### 3.1. General Features of the Study Cohort

All the 71 patients screened from the two cohorts had complete data and usable MRIs for the analysis. The mean age was 70.2 years, and 19 (26.8%) subjects were female. A history of hypertension, hyperlipidemia, or diabetes mellitus was common (66%, 55%, and 32%, respectively). Table 1 shows complete patient demographic, clinical, and imaging features. The two cohorts showed similar features except for female sex frequency, number of cigarettes/day, and number of enlarged PVS in the white matter (Table 1).

According to the visual evaluation of PVS, the median (IQR) score was two (2–3) for the basal ganglia and white matter regions. Grade 1 in the midbrain was found in 53 (74.7%) patients.

**Figure 2:**
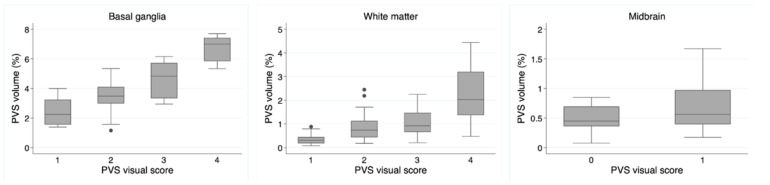
Box plot analysis between visual PVS evaluation and computed PVS measures. Values in the *y*-axis represent the fractional PVS volume (%) calculated in the 3 brain locations (basal ganglia, white matter, and brainstems), while the *x*-axis represents the visual PVS scores in the same regions.

There was a good correlation (Rho, *p*) between PVS visual scores and quantitative segmentations in the basal ganglia (0.72, <0.001) and white matter (0.65, <0.001) regions, while it was not significant in the midbrain (0.20, 0.088), as shown in the boxplots (Figure 2).

**Figure 3:**
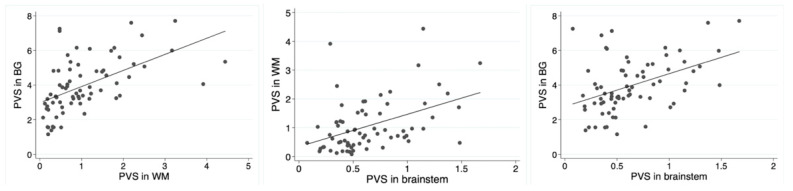
Scatter point analysis between PVS measures in different locations. The graphs show the linear relationships between the fractional volumes of PVS in the three brain locations (basal ganglia, white matter, and brainstems).

The fractional volumes of PVS from the three brain regions were related to each other, with a Pearson’s correlation coefficient, respectively, of 0.54 between the WM and BG, 0.44 between the WM and brainstem, and 0.43 between the basal ganglia and brainstem (*p* < 0.001 in all cases). The distribution of these correlations had a linear shape with an R square of 0.29, 0.19, and 0.19, respectively (Figure 3).

### 3.2. Univariable and Multivariable Regression Analyses

Since the fractional PVS volume did not have a normal distribution, we tested nine different variable transformation algorithms, choosing the one that fitted better for the three regions where PVS were quantified, which was logarithmic transformation. The complete normality analyses are available in the Supplementary Figure S1. We adopted the normalized fractional PVS volumes as dependent variables for all linear regression analyses.

### 3.3. Analyses Using PVS Fractional Volumes in Basal Ganglia as the Dependent Variable

In the linear regression analyses, age (*p* < 0.001), arterial hypertension (*p* = 0.001), number of lacunes (*p* = 0.017), number of microbleeds (*p* = 0.046), and WMH volume (*p* = 0.001) were associated with enlarged PVS in the basal ganglia in the unadjusted model. After introducing age, sex, and hypertension as covariates in the model, the number of lacunes (*p* = 0.034), number of microbleeds (*p* = 0.006), and WMH volume (0.017) were independently associated with enlarged PVS in the basal ganglia (Table 2).

### 3.4. Analyses Using PVS Fractional Volume in White Matter as the Dependent Variable

In the unadjusted model, age (*p* = 0.013), arterial hypertension (*p* = 0.001), and the number of lacunes (*p* = 0.001) were associated with the PVS volume in the WM; in the model adjusted for age and hypertension, only the number of lacunes (*p* = 0.003) were independently associated with enlarged PVS in the white matter (Table 2).

### 3.5. Analyses Using the PVS Fractional Volume in the Brainstem as the Dependent Variable

In the unadjusted analysis, only the presence of diabetes mellitus was associated with a higher PVS volume in the brainstem (*p* = 0.041). However, this association was no longer significant after adjusting for age, sex, and hypertension (*p* = 0.235) (Table 2).

### 3.6. Analyses Using PVS Ratios Between the White Matter and Basal Ganglia as the Dependent Variable

In the unadjusted analysis using the WM/BG-PVS ratio as the dependent variable, arterial hypertension (*p* = 0.034), the number of lacunes (*p* = 0.028), and the PSQI score (*p* = 0.010) were associated with a higher ratio. In the adjusted analysis for age, sex, and hypertension, the number of lacunes (*p* = 0.018) and the PSQI score (*p* = 0.046) maintained a significant association (Table 2).

## 4. Discussion

In this cohort of patients with ischemic strokes related to SVD, there was a consistent correlation between the load of dilated PVS in different brain regions. However, the number of PVS in specific brain regions showed varying associations with other SVD imaging markers and sleep quality. This suggests that different physiological processes, such as different hemodynamic conditions and the clearance of fluids, may have distinct effects in different brain regions.

The presence of dilated PVS was common in all brain regions in patients with an RSSI. The fractional PVS volume from different areas was highly correlated and showed a linear trend. Our analysis confirmed that the number of PVS depended on the presence of other markers of SVD, especially lacunes, WMHs, and microbleeds, independently of age and history of hypertension. However, the relationships varied based on the regions. For instance, we found a significant association between WMH volume and fractional PVS volume in the BG, but not in the WM or BS. This varying topographic correlation between WMHs and PVS was previously described in other studies, including a cohort of 215 patients with an RSSI [17], a cohort of 530 population-based individuals older than 60 years [18], a cohort of 296 with similar characteristics [19], and a cohort of 1575 population-based subjects [20]. On the contrary, another study showed a similar correlation between PVS visually graded in the basal ganglia and centrum semiovale and WMH volume [21]. However, this study was conducted on a cohort of 887 individuals younger than 45 years who underwent an MRI for several reasons, in which the presence of WMHs could be related to mechanisms other than SVD, such as in the case of migraines. Several different mechanisms involving PVS enlargement could explain these results. First, arterioles in the basal ganglia and centrum semiovale are anatomically different, since the formers have two leptomeningeal membranes, allowing direct communication between the perivascular and subarachnoid spaces, while the latter have only one leptomeningeal membrane, connecting the perivascular and subpial spaces [4]. These different anatomical features, along with different pressure gradients and pulsatility, may explain why arterioles in the basal ganglia might be more prone to hypertensive injury leading to arterial stiffness and PVS enlargement than those in the WM [22,23]. In addition, long-term hypoperfusion in the centrum semiovale in SVD and vascular reduction in WMHs [24] might also contribute to reduced visibility of enlarged PVS [25]. However, longitudinal studies untangling the relationships between PVS and WMH formation are warranted, which are challenging since they require several years of follow-up to detect significant changes.

As regards the number of lacunes, there was a strong association with PVS load in the BG and WM, which is a known association, as described in a recent pooled analysis including 39,976 individuals from 10 population-based cohort studies [26]. Unexpectedly, patients with a higher WM/BG-PVS ratio had a greater number of lacunes, even after adjusting for covariates. This association could be due to severe WM disease leading to multiple cavitations, despite a relatively low involvement in the BG. It is also possible that enlarged PVS were misclassified as lacunes, as we used the pragmatic STRIVE criteria to minimize subjective evaluation by raters. Nevertheless, the PVS ratio is a new method for examining regional variations and could be utilized in other cohorts to validate these results in the future.

Our study results indicated a potential connection between PVS patterns and sleep quality. There was a positive trend between PVS load and the PSQI in the WM, but not in the BG or BS. This association was apparent when analyzing the ratio between PVS volume and WM and BG, showing significant associations in both unadjusted and adjusted models. While PVS enlargement tends to affect lobar and deep-brain regions proportionally, the higher fractional volume in the white matter may signify disrupted brain fluid clearance, particularly during sleep. Numerous studies have investigated the link between PVS and sleep by analyzing the total PVS volume or examining them separately in various brain regions, showing controversial results [9,27,28]. We need further investigation to understand the intricate connections between PVS dilation, the glymphatic system, and sleep disorders. In addition to requiring more accurate methods to assess sleep quality and effectiveness, like wearable sleep-tracking devices and polysomnography, examining the ratio of PVS in different brain regions could uncover differences in underlying mechanisms.

The analysis of PVS in the brainstem showed no significant associations with clinical and imaging variables. It is possible that because this region is smaller than the others, the study was underpowered to find significant associations. Additionally, the correlation between the visual scale and the volumetric PVS values was poor, likely due to the different areas of the brainstem included in the PVS segmentation (entire brainstem) and the visual scale (only midbrain).

The study had several strengths. Firstly, the imaging protocol used a 3T MRI according to the HARmoNising Brain Imaging MEthodS for VaScular Contributions to Neurodegeneration (HARNESS) recommendations for SVD research [10]. Secondly, despite the study’s post hoc design, all the study variables used for the analysis were collected prospectively. Thirdly, we employed objective measures of PVS volume using a validated PVS segmentation method, which was further improved by manual correction.

The study also has a few limitations. Firstly, we acknowledge the small sample size of the cohort, which may limit the ability to detect significant differences between groups. However, this limitation is balanced by the fact that the patients in the study had similar characteristics and the same imaging and acquisition protocol. It is important to note that the patients were from two different cohorts, but we ensured that their clinical or imaging features were comparable. On top of that, we are planning to confirm the results of the present study in a larger cohort of patients also including diffusion-tensor imaging (DTI) measures, such as DTI along the PVS (DTI-ALPSs) and free-water fraction, for the assessment of brain fluid content and clearance. Finally, the sleep quality questionnaires used in the study were subjective and may have been less reliable in cases of cognitive impairment. Unfortunately, we were unable to conduct objective sleep tests, such as polysomnography or home-actigraphy. Nonetheless, the questionnaire can provide an estimate of sleep quality over extended periods and may be useful for exploratory research purposes.

## 5. Conclusions

This pilot study examined the regional characteristics of PVS in patients with an RSSI, a common marker of SVD. The study highlighted that while there is an overall association between PVS load in the WM, BG, and BS, regional differences may indicate distinct SVD patterns. Additionally, more pronounced PVS involvement in the WM as opposed to deep regions was linked to poorer sleep quality, suggesting impaired brain fluid clearance. These findings should be validated in larger groups of patients with similar characteristics and compared to other populations.

## Figures and Tables

**Table 1:** Clinical, demographic, and radiological characteristics of the study population.

Clinical Characteristics	All N = 71	Cohort 1 N = 50	Cohort 2 N = 21
Age, years, mean (SD)	70.2 (10.8)	70.1 (11.5)	70.6 (9.2)
Female sex, *n* (%)	19 (26.8)	18 (36.0)	1 (4.8)
Arterial hypertension, *n* (%)	47 (66.2)	31 (62.0)	16 (76.2)
Hyperlipidemia, *n* (%)	39 (54.9)	29 (58.0)	10 (47.6)
Diabetes mellitus, *n* (%)	23 (32.4)	17 (34.0)	6 (28.6)
Any smoking, *n* (%)	24 (33.8)	18 (36.0)	6 (28.6)
Any alcohol intake, *n* (%)	28 (39.4)	20 (40.0)	8 (38.1)
PSQI, median (IQR)	6 (4–10)	6 (4–10)	7 (4.5–10)
ESS score, median (IQR)	5 (2–7)	5 (2–7)	6 (2.5–7)
Lacunes, presence of, *n* (%)	1.9 (2.5)	33 (66.0)	11 (52.4)
Lacunes, number of, median (IQR)	1 (0–3)	1 (0–3)	1 (0–3)
Cerebral microbleeds, presence of, *n* (%)	18 (25.35)	11 (22.0)	7 (33.3)
Cerebral microbleeds, number, median (IQR)	0 (0–0)	0 (0–0)	0 (0–1.5)
BG-PVS, percentage, mean (SD)	4.0 (1.6)	0.89 (0.78)	1.48 (1.07)
WM-PVS, percentage, mean (SD)	1.1 (0.9)	0.66 (0.38)	0.57 (0.30)
BS-PVS, percentage, mean (SD)	0.6 (0.4)	0.66 (0.38)	0.57 (0.30)
Fazekas score, periventricular areas, median (IQR)	2 (1–2)	2 (1–2)	2 (1–2)
Fazekas score, deep white matter, median (IQR)	1 (1–2)	1 (1–2)	1 (1–2)
WMH ratio (%), mean (SD)	1.1 (1.0)	1.2 (1.0)	1.0 (1.0)
NIHSS, median (IQR)	2.5 (1–4)	3 (1–5)	2 (1–4)

PSQI: Pittsburgh Sleep Quality Index; ESS: Epworth Sleepiness Scale; BG: basal ganglia; WM: white matter; BS: brainstem; PVS: perivascular space; WMH ratio: white matter hyperintensities/intracranial volume; NIHSS: National Institute of Health Stroke Scale.

**Table 2:** Simple and multiple linear regression models (adjusted for age, sex, and hypertension).

Dependent Variable: BG-PVS % (log)	Unadjusted Model			Adjusted Model		
Regressors:	βs^−^	*p*	R^2^ (%)	βs^−^	*p*	R^2^ (%)
Age	0.022	<0.001 *	29.4	0.018	<0.001 *	36.8
Female sex	0.050	0.668	0.3	−0.008	0.934	36.84
Arterial hypertension	0.363	0.001 *	16.1	0.256	0.007 *	36.84
Dyslipidemia	0.049	0.634	0.3	−0.085	0.337	37.7
Diabetes mellitus	0.134	0.222	2.2	−0.092	0.351	37.7
Body Mass Index	−0.0009	0.514	0.6	−0.001	0.384	37.8
Any smoking	−0.130	0.231	2.1	0.052	0.587	37.1
Any alcohol intake	−0.088	0.403	1.0	−0.011	0.900	36.9
PSQI	−0.0003	0.979	0.0	−0.006	0.581	38.9
ESS score	−0.002	0.888	0.0	0.009	0.494	38.8
Number of lacunes	0.049	0.017 *	8.0	0.037	0.034 *	41.0
Number of cerebral microbleeds	0.053	0.046 *	5.7	0.061	0.006 *	44.2
WMH ratio	0.162	0.001 *	14.9	0.109	0.017 *	42.2
Dependent variable: WM-PVS % (log)	Unadjusted model			Adjusted model		
Regressors:	βs^−^	*p*	R^2^ (%)	βs^−^	*p*	R^2^ (%)
Age	0.024	0.013 *	8.6	0017	0.073	20.8
Female sex	−0.096	0.689	0.2	−0.226	0.308	20.8
Arterial hypertension	0.735	0.001 *	15.5	0.673	0.002 *	20.8
Dyslipidemia	0.298	0.162	2.8	0.068	0.742	20.9
Diabetes mellitus	0.245	0.280	1.7	−0.151	0.508	21.3
Body Mass Index	−0.0009	0.765	0.1	−0.002	0.565	21.1
Any smoking	0.042	0.853	0.0	0.314	0.150	23.4
Any alcohol intake	0.090	0.681	0.2	0.175	0.387	21.7
PSQI	0.045	0.086	4.5	0.035	0.144	24.8
ESS score	−0.020	0.531	0.6	−0.007	0.793	20.9
Number of lacunes	0.135	0.001 *	14.4	0.117	0.003 *	30.8
Number of cerebral microbleeds	0.095	0.085	4.3	0.086	0.098	24.0
WMH ratio	0.159	0.126	3.4	0.089	0.405	21.6
Dependent variable: BS-PVS % (log)	Unadjusted model			Adjusted model		
Regressors:	βs^−^	*p*	R^2^ (%)	βs^−^	*p*	R^2^ (%)
Age	0.012	0.076	4.5	0.010	0.151	8.5
Female sex	−0.164	0.305	1.5	−0.206	0.196	8.5
Arterial hypertension	0.223	0.135	3.2	0.198	0.197	8.5
Dyslipidemia	0.135	0.343	1.3	0.063	0.667	8.7
Diabetes mellitus	0.306	0.041 *	5.9	0.194	0.235	10.4
Body Mass Index	−0.001	0.460	0.8	−0.002	0.362	9.8
Any smoking	−0.049	0.743	0.1	0.079	0.616	8.8
Any alcohol intake	−0.045	0.755	0.1	−0.030	0.834	8.5
PSQI	0.019	0.269	1.9	0.018	0.295	10.4
ESS score	0.037	0.861	0.0	0.006	0.788	8.9
Number of lacunes	0.007	0.481	0.7	0.017	0.570	8.9
Number of cerebral microbleeds	0.025	0.499	0.7	0.026	0.494	9.0
WMH ratio	0.085	0.218	2.2	0.090	0.242	10.4
Dependent variable: WM/BG-PVS % (log)	Unadjusted model			Adjusted model		
Regressors:	βs^−^	*p*	R^2^ (%)	βs^−^	*p*	R^2^ (%)
Age	0.002	0.751	0.15	−0.002	0.808	8.3
Female sex	−0.146	0.441	0.86	−0.218	0.248	8.3
Arterial hypertension	0.373	0.034 *	6.38	0.417	0.024 *	8.3
Dyslipidemia	0.248	0.139	3.14	0.153	0.381	9.4
Diabetes mellitus	0.111	0.538	0.55	−0.060	0.761	8.4
Body Mass Index	0.000	0.983	0.00	−0.001	0.818	8.2
Any smoking	0.172	0.333	1.36	0.263	0.157	11.1
Any alcohol intake	0.178	0.301	1.55	0.186	0.279	9.93
PSQI	0.046	0.028 *	7.36	0.041	0.046 *	14.4
ESS score	−0.017	0.480	0.74	−0.016	0.514	8.7
Number of lacunes	0.086	0.010 *	9.35	0.080	0.018 *	15.8
Number of cerebral microbleeds	0.042	0.338	1.35	0.025	0.571	9.1
WMH ratio	−0.003	0.967	0.00	−0.020	0.828	8.4

BG: basal ganglia; WM: white matter; BS: brainstem; PVS: perivascular space; PSQI: Pittsburgh Sleep Quality Index; ESS: Epworth Sleepiness Scale; WMH ratio: white matter hyperintensities/intracranial volume. * *p* < 0.05.

## Data Availability

Data not provided in the article are available upon reasonable request.

## Supplementary Materials

The following supporting information can be downloaded at: https://www.mdpi.com/article/10.3390/jcdd11110345/s1, Figure S1: Normality analysis by histograms of perivascular space (PVS) fractional volumes.

## Abbreviations

Perivascular spaces—PVS; small vessel disease—SVD; recent small subcortical infarct—RSSI; Pittsburg Sleep Quality Index—PSQUI; PVS in white matter—WM-PVS; PVS in basal ganglia—BG-PVS; PVS in brainstem—BS-PVS; white matter hyperintensities—WMHs; diffusion-tensor imaging along the perivascular spaces—DTI-ALPSs; Standards for ReportIng Vascular changes on nEuroimaging—STRIVE; National Institute of Health Stroke Scale—NIHSS; normal-appearing white matter—NAWM.

## References

[1] Pasi M., Cordonnier C. (2020). Clinical Relevance of Cerebral Small Vessel Diseases. Stroke.

[2] Lam B.Y.K., Cai Y., Akinyemi R., Biessels G.J., van den Brink H., Chen C., Cheung C.W., Chow K.N., Chung H.K.H., Duering M. (2023). The global burden of cerebral small vessel disease in low- and middle-income countries: A systematic review and meta-analysis. Int. J. Stroke.

[3] Duering M., Biessels G.J., Brodtmann A., Chen C., Cordonnier C., de Leeuw F.E., Debette S., Frayne R., Jouvent E., Rost N.S. (2023). Neuroimaging standards for research into small vessel disease-advances since 2013. Lancet Neurol..

[4] Wardlaw J.M., Benveniste H., Nedergaard M., Zlokovic B.V., Mestre H., Lee H., Doubal F.N., Brown R., Ramirez J., MacIntosh B.J. (2020). Perivascular spaces in the brain: Anatomy, physiology and pathology. Nat. Rev. Neurol..

[5] Rodriguez Lara F., Toro A.R., Pinheiro A., Demissie S., Ekenze O., Martinez O., Parva P., Charidimou A., Ghosh S., DeCarli C. (2023). Relation of MRI-Visible Perivascular Spaces and Other MRI Markers of Cerebral Small Vessel Disease. Brain Sci..

[6] Francis F., Ballerini L., Wardlaw J.M. (2019). Perivascular spaces and their associations with risk factors, clinical disorders and neuroimaging features: A systematic review and meta-analysis. Int. J. Stroke.

[7] Malhotra K., Theodorou A., Katsanos A.H., Zompola C., Shoamanesh A., Boviatsis E., Paraskevas G.P., Spilioti M., Cordonnier C., Werring D.J. (2022). Prevalence of Clinical and Neuroimaging Markers in Cerebral Amyloid Angiopathy: A Systematic Review and Meta-Analysis. Stroke.

[8] Wang X.X., Cao Q.C., Teng J.F., Wang R.F., Yang Z.T., Wang M.G., Cao Z.-H. (2022). MRI-visible enlarged perivascular spaces: Imaging marker to predict cognitive impairment in older chronic insomnia patients. Eur. Radiol..

[9] Dredla B.K., Del Brutto O.H., Castillo P.R. (2023). Sleep and Perivascular Spaces. Curr. Neurol. Neurosci. Rep..

[10] Smith E.E., Biessels G.J., De Guio F., de Leeuw F.E., Duchesne S., Düring M., Frayne R., Ikram M.A., Jouvent E., MacIntosh B.J. (2019). Harmonizing brain magnetic resonance imaging methods for vascular contributions to neurodegeneration. Alzheimers Dement..

[11] Staals J., Makin S.D.J., Doubal F.N., Dennis M.S., Wardlaw J.M. (2014). Stroke subtype, vascular risk factors, and total MRI brain small-vessel disease burden. Neurology.

[12] Potter G.M., Chappell F.M., Morris Z., Wardlaw J.M. (2015). Cerebral perivascular spaces visible on magnetic resonance imaging: Development of a qualitative rating scale and its observer reliability. Cerebrovasc. Dis..

[13] Barnes A., Ballerini L., Valdés Hernández M.d.C., Chappell F.M., Muñoz Maniega S., Meijboom R., Backhouse E.V., Stringer M.S., Coello R.D., Brown R. (2022). Topological relationships between perivascular spaces and progression of white matter hyperintensities: A pilot study in a sample of the Lothian Birth Cohort 1936. Front Neurol.

[14] Valdés Hernández M.D.C., Ballerini L., Glatz A., Aribisala B.S., Bastin M.E., Dickie D.A., Duarte C.R., Munoz M.S., Wardlaw J.M. (2023). Step-by-Step Pipeline for Segmenting Enlarged Perivascular Spaces from 3D T2-Weighted MRI, 2018–2023.

[15] Duarte Coello R., Valdés Hernández M.d.C., Zwanenburg J.J.M., van der Velden M., Kuijf H.J., De Luca A., Moyano J.B., Ballerini L., Chappell F.M., Brown R. (2024). Detectability and accuracy of computational measurements of in-silico and physical representations of enlarged perivascular spaces from magnetic resonance images. J. Neurosci. Methods.

[16] Valdés Hernández M.D.C., Ferguson K.J., Chappell F.M., Wardlaw J.M. (2010). New multispectral MRI data fusion technique for white matter lesion segmentation: Method and comparison with thresholding in FLAIR images. Eur. Radiol..

[17] Jian X., Xu F., Yang M., Zhang M., Yun W. (2023). Correlation between enlarged perivascular space and brain white matter hyperintensities in patients with recent small subcortical infarct. Brain Behav..

[18] Laveskog A., Wang R., Bronge L., Wahlund L.O., Qiu C. (2018). Perivascular Spaces in Old Age: Assessment, Distribution, and Correlation with White Matter Hyperintensities. AJNR Am. J. Neuroradiol..

[19] Yamada S., Ishikawa M., Yamamoto K., Yamaguchi M., Oshima M. (2019). Location-specific characteristics of perivascular spaces as the brain’s interstitial fluid drainage system. J. Neurol. Sci..

[20] Yakushiji Y., Charidimou A., Hara M., Noguchi T., Nishihara M., Eriguchi M., Nanri Y., Nishiyama M., Werring D.J., Hara H. (2014). Topography and associations of perivascular spaces in healthy adults: The Kashima scan study. Neurology.

[21] Zou Q., Wang M., Zhang D., Wei X., Li W. (2023). White Matter Hyperintensities in Young Patients from a Neurological Outpatient Clinic: Prevalence, Risk Factors, and Correlation with Enlarged Perivascular Spaces. J. Pers. Med..

[22] Blanco P.J., Müller L.O., Spence J.D. (2017). Blood pressure gradients in cerebral arteries: A clue to pathogenesis of cerebral small vessel disease. Stroke Vasc. Neurol..

[23] Riba-Llena I., Jiménez-Balado J., Castañé X., Girona A., López-Rueda A., Mundet X., Jarca C.I., Álvarez-Sabin J., Montaner J., Delgado P. (2018). Arterial Stiffness Is Associated With Basal Ganglia Enlarged Perivascular Spaces and Cerebral Small Vessel Disease Load. Stroke.

[24] Shi Y., Thrippleton M.J., Makin S.D., Marshall I., Geerlings M.I., De Craen A.J.M., van Buchem M.A., Wardlaw J.M. (2016). Cerebral blood flow in small vessel disease: A systematic review and meta-analysis. J. Cereb. Blood Flow Metab..

[25] Wang H., Nie Z.Y., Liu M., Li R.R., Huang L.H., Lu Z., Jin L.-J., Li Y.-X. (2020). Clinical characteristics of perivascular space and brain CT perfusion in stroke-free patients with intracranial and extracranial atherosclerosis of different extents. Ann. Transl. Med..

[26] Evans T.E., Knol M.J., Schwingenschuh P., Wittfeld K., Hilal S., Ikram M.A., Dubost F., van Wijnen K.M.H., Katschnig P., Yilmaz P. (2023). Determinants of Perivascular Spaces in the General Population: A Pooled Cohort Analysis of Individual Participant Data. Neurology.

[27] Lysen T.S., Yilmaz P., Dubost F., Ikram M.A., de Bruijne M., Vernooij M.W., Luik A.I. (2022). Sleep and perivascular spaces in the middle-aged and elderly population. J. Sleep Res..

[28] Shih N.C., Barisano G., Lincoln K.D., Mack W.J., Sepehrband F., Choupan J. (2023). Effects of sleep on brain perivascular space in a cognitively healthy population. Sleep Med..

