# Supplementary figures and images for: Clinical Relevance of Different Loads of Perivascular Spaces According to Their Localization in Patients with a Recent Small Subcortical Infarct
Source: J Cardiovasc Dev Dis. 2024 Nov 1;11(11):345. doi: 10.3390/jcdd11110345 (PMC11594638; doi:10.3390/jcdd11110345)

**Figure S1: Normality analysis by histograms of perivascular space (PVS) fractional volumes**

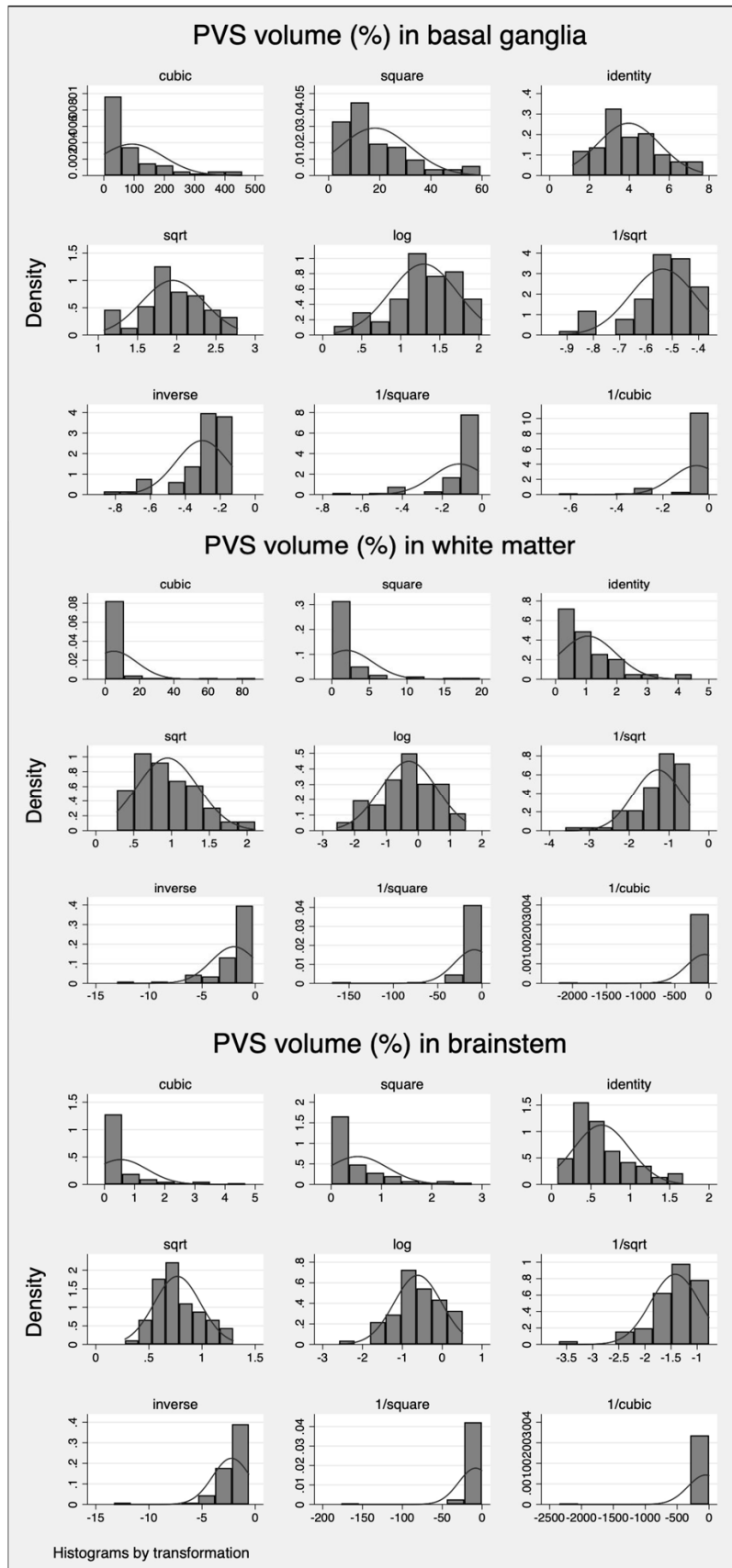

Supplement: Supplementary file 1 [file jcdd-11-00345-s001.zip › jcdd-3255102-supplementary.pdf]
